# Insecticidal and Histopathological Effects of *Ageratum conyzoides* Weed Extracts against Dengue Vector, *Aedes aegypti*

**DOI:** 10.3390/insects11040224

**Published:** 2020-04-03

**Authors:** Ai-rada Pintong, Sumate Ampawong, Narumon Komalamisra, Patchara Sriwichai, Supaluk Popruk, Jiraporn Ruangsittichai

**Affiliations:** 1 Department of Medical Entomology, Faculty of Tropical Medicine, Mahidol University, Ratchawithi Road, Ratchathewi, Bangkok 10400, Thailand; aeuyrada2015@gmail.com (A.-r.P.); narumon.kom@mahidol.ac.th (N.K.); patchara.sri@mahidol.ac.th (P.S.); 2 Department of Tropical Pathology, Faculty of Tropical Medicine, Mahidol University, Ratchawithi Road, Ratchathewi, Bangkok 10400, Thailand; am_sumate@hotmail.com; 3 Department of Protozoology, Faculty of Tropical Medicine, Mahidol University, Ratchawithi Road, Ratchathewi, Bangkok 10400, Thailand; supaluk.pop@mahidol.ac.th

**Keywords:** Asteraceae, *Aedes*, essential oils, crude extracts, insecticide, pesticide, vector control

## Abstract

Crude extracts and essential oils of *A. conyzoides* were tested with larva and adult stages of *Ae. aegypti* mosquitoes to determine their insecticidal properties. The crude extracts and essential oils came from three varieties of *A. conyzoides* (with white flowers, purple flowers, or white-purple flowers) and from two places on each plant (leaves and flowers), giving six types overall: leaf-white (LW); leaf-purple (LP); leaf white-purple (LW-P); flower-white (FW); flower-purple (FP); and flower white-purple (FW-P). Chemical constituents and components of the essential oils were identified using gas chromatography-mass spectrometry (GC-MS). Electron microscopic and histopathological studies were performed to determine the toxicological effects on mosquitoes in terms of morphological alterations. The six types of crude extracts exhibited no activity against individuals in the larval stages. However, six types of essential oils were effective against adult *Ae. aegypti* females. The mortality of adult *Ae. aegypti* females was higher from leaf extracts, particularly LP (median lethal dose, LD_50_ = 0.84%). The number of chemical constituents identified by GC-MS was high in flowers, especially W-P. Precocene I was the most abundant chemical component among the five types of essential oils, except in LP, in which precocene II was the most abundant. Histopathological alterations in adult *Ae. aegypti* females included compound eye degeneration, muscular damage with cellular infiltration, gut epithelial degeneration and necrosis, pyknotic nuclei in the malpighian epithelium and ovarian cell degeneration. FW and FP plant types exhibited the highest severity of histopathological alterations in mosquitoes compared with other plants, probably owing to the presence of monoterpene compounds in their tissues. The present study demonstrated LP plant extracts from *A*. *conyzoides* could be effective adulticides against adult *Ae*. *aegypti*. As natural products are biodegradable and exhibit low toxicity to mammalian and non-target organisms, they are suitable candidates for use in vector control programmes.

## 1. Introduction

Dengue is currently widespread throughout subtropical and tropical regions. The incidence of dengue infections worldwide is approximately 390 million people every year [1]. Over 50% of the world’s population resides in areas with risk of infection, and approximately 50% resides in endemic regions [2,3]. *Aedes aegypti* is the major mosquito vector. Although vector control programmes largely depend on the use of synthetic insecticides, which has been sustained over many decades, consistently using synthetic pesticides could lead to ineffective control due to the development of insecticide resistance in *Ae. aegypti* in many countries worldwide, such as Indonesia, India, Malaysia, Thailand, China, Mexico, Columbia and Brazil [4,5].

Therefore, it is necessary to urgently explore alternative control measures. One of the alternative ways to control larva and adult mosquitoes is using natural plant-derived products as pesticide candidates and enhancing the efficacy of chemical pesticide [6,7]. Such products are non-persistent and non-toxic to humans and have shorter environmental persistence than synthetic insecticides, in addition to varying sites of action, which have not been previously reported in mosquito resistance [8,9].

*A. conyzoides* is an annual herbaceous plant with worldwide distribution, particularly in the subtropical and tropical regions, which belongs to the family *Asteraceae* [10]. The genus *Ageratum* comprises 30 species. However, only a few species have been observed to exhibit phytochemical activity [11]. *A. conyzoides* exhibits high morphological variation. For example, the flowers may have different colours, ranging from white to purple. Additionally, the plant is easily adaptable to different ecological conditions [12]. It is a weed that has invaded agricultural and grazing areas. It has also been utilised in traditional medicine as a purgative, a febrifuge, an anti-ulcer medication and for wound dressing in Africa, as a bactericide, an anti-dysenteric and an anti-lithic in India, to treat fever measles and snake bites in Togo, and for wound healing, treating diarrhoea and relieving pain associated with navels in children in Nigeria [10,13,14]. There are diverse phytochemicals in the secondary metabolites of *A. conyzoides*, with terpenoids being the major components [15]. Many of the metabolites are biologically active, for example, with anti-parasitic, anti-inflammatory, anti-coagulant, myorelaxant, haemostatic, analgesic, anti-fungal and hypothermic activity. In addition, *A. conyzoides* has been demonstrated to exhibit insecticidal activity, which could be the most significant biological activity in the species [16,17,18]. Numerous researchers have investigated *A. conyzoides* plant extracts, such as crude extracts and essential oils, using different solvents and methods of distillation from whole plants, leaves, stems, roots, barks and aerial parts against larva or adult stages of mosquitoes. According to the results of these studies, the plant extracts exhibit potential activity against disease vectors.

As no studies have been performed on the relative toxicity of efficiency of *A. conyzoides* extracts, six types of crude extracts and essential oils from leaf (L) and flower (F) parts from white (W), purple (P) and white-purple (W-P) flowers or mixed colours were tested for their efficiency against *Ae. aegypti* larval and adult stages. Numerous chemical profiles of *A. conyzoides* have been documented. However, there is still no information on the chemical components of leaves and white, purple and white-purple coloured flower parts, as well as the relative toxicities of the six types of essential oils to the external and internal organs of adult mosquitoes. Therefore, the present study aimed to investigate the efficiency of crude extracts and essential oils from each of the plant’s parts and from flowers of different colours regarding the larvicidal and adulticidal activity, respectively. The present study aimed to provide data on the chemical composition of six types of essential oils through gas chromatography-mass spectrometry (GC-MS) studies. Histopathological and electron microscopic studies were performed to observe morphological changes in the mosquitoes following treatment or non-treatment with the six types of essential oils. This study presents the first investigation of *Ae. aegypti* mosquitoes wherein the effects of six types of both crude extracts and essential oils from *A*. *conyzoides* on both larval and adult stages were tested. We analysed the chemical constituents of the essential oils and investigated their toxicological effects on mosquitoes. The results provide information that could facilitate the control of *Ae. aegypti* mosquitoes using alternative methods and reveal the toxicity of chemical constituents of the extracts on mosquito morphology.

## 2. Materials and Methodology

### 2.1. Collection of Plants

**Figure 1:**
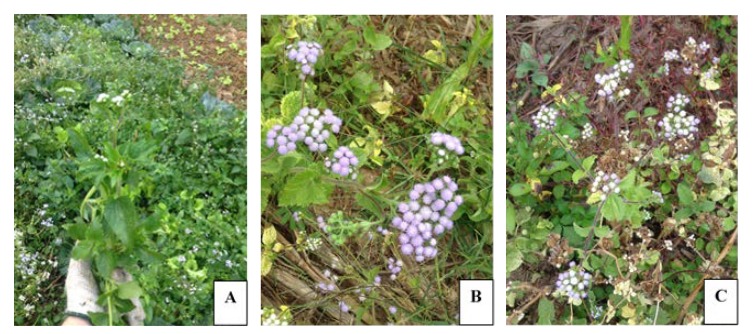
*A. conyzoides* weeds were collected from Chiang Rai province: white flower plant (**A**), purple flower plant (**B**), white-purple flower plant (**C**).

*A. conyzoides* were collected from wastelands in Chiang Kong district, Chiang Rai province (20°15′36″ N 100°24′24″ E), Thailand. Plants were collected in period March 2016– March 2017. At the site of collection, the authors observed differently-coloured flower parts in each plant, including white, purple and white-purple colours, which were harvested and separated to facilitate identification of the plant species at the Department of Botany, Faculty of Science, Chulalongkorn University. The voucher specimens, numbered 015854, were deposited in Professor Kasin Suvatabhandhu Herbarium, Department of Botany, Faculty of Science, Chulalongkorn University. The results showed that the plants exhibiting three different flower colours were the same species, *A. conyzoides*. Six types of crude extracts and essential oils were used for the investigations in the present study, which leaf-white (LW); leaf-purple (LP); leaf white-purple (LW-P); flower-white (FW); flower-purple (FP); and flower white-purple (FW-P), as shown in Table 1 and Figure 1. Plants were taken to the Faculty of Tropical Medicine, Mahidol University.

### 2.2. Plant Extraction

#### 2.2.1. Crude Extractions

Each part of the fresh plants was dried at 60 °C in an oven for 1 week. They were kept in plastic bags and stored in a dry and cool place. Six samples of dried plant material were pulverised prior to extraction. For each extraction procedure, powdered plant material was macerated using absolute ethanol (EtOH) and the extract was filtered using Whatman paper (0.45 µm in diameter). After filtration, the solvent was obtained using a rotary evaporator (Heidolph, Germany). The concentrates were dried and stored at 4 °C until further use.

#### 2.2.2. Essential Oils

Leaf and flower parts of the fresh plants were cut into small pieces using a grinding mill, suspended in distilled water and subjected to hydrodistillation for 3 h. Essential oils appeared at the top of the pipette, which was connected to the condenser. To remove any traces of water, sodium sulphate (Na_2_SO_4_) was used. The essential oils were stored in dark clean glass vials at 4 °C until used.

### 2.3. Rearing Mosquitoes

Larvicidal and adulticidal activity in mosquitoes were evaluated using laboratory-reared *Ae. aegypti* Bora (French Polynesia) strains domesticated in the Department of Medical Entomology, Faculty of Tropical Medicine, Mahidol University. Mosquito eggs were maintained in an insectarium. The eggs were placed in dechlorinated water to hatch. The larvae were reared in plastic containers without exposure to any insecticide. Each container contained 150 larvae for 1 L of dechlorinated water. The emerging larvae were fed with fish powder [19]. The emerging adults were fed a 5% sugar solution by placing a soggy cotton wool ball in cages. The average temperature inside the insectarium was maintained at 28 °C ± 2 °C with a relative humidity of 75% ± 5%.

### 2.4. Mosquito Testing

#### 2.4.1. Larvicidal Activity

There were six types of crude extracts from *A. conyzoides*. Each crude extract was dissolved in dimethyl sulfoxide (DMSO) to prepare 1% *w/v* (10,000 mg/L) stock solution. At first, the crude extracts to be screened for effectiveness were prepared in concentrations of 0.001% (10 mg/L) [20]. Meanwhile, two control tests were set up for comparison: one comprised of distilled water and another comprised of DMSO. Each treatment had 20 early fourth instar larvae of *Ae. aegypti* Bora (French Polynesia) strain. There were four replicates for both the treatments and controls. Mortality was recorded after 24 and 48 h of the experiments. Dead larvae were identified by lack of movement, discoloration, unnatural positions, incoordination, or rigour.

#### 2.4.2. Adulticidal Activity

Adulticidal activity was investigated by topical application on the adult female *Ae. aegypti* Bora (French Polynesia) strain to investigate susceptibility to the extracts. Each essential oil was dissolved in acetone before testing on mosquitoes. Females not fed on blood were anaesthetised for 25–60 s with a vapour of ether in a bottle and gently arranged using forceps on a plate. Two sets of experiments were arranged. The first set included controls, the acetone treatment and the untreated groups. The second set involved treatments with essential oils in acetone. Each concentration of essential oil in acetone was tested against 60 mosquitoes, with seven concentrations with 0.70–2.00%, 0.40–1.95% and 0.55–1.85% in essential oil types LW, LP and LW-P, respectively, and 0.70–2.00%, 0.45–1.95% and 0.55–1.85% in essential oils types FW, FP and FW-P, respectively, providing a range of 10–90% mortality; controls were run concurrently. Exposures to all sets were performed in triplicate. Essential oil solutions in 0.5 µL acetone were dropped on the thorax of mosquitoes using topical applicators. After applying the essential oils on the mosquitoes, they were transferred to slightly damp plastic cups and covered with mesh fabric lids with rubber bands. Additionally, 5% sucrose saturated cotton roll was secured at the top of the mesh fabric. Mortality was recorded at 24 h post treatment. The mosquitoes were considered dead if they did not move at the bottom of the plastic cups and respond to mechanical stimulation.

### 2.5. GC-MS Analysis

Six types of essential oils were subjected to GC-MS analysis for the identification of constituents and components. The GC-MS analysis was conducted on an Agilent Technologies 6980N GC chromatograph, equipped with a HP-5 MS capillary column (30 m × 0.25 mm × 0.25 µm) and a mass spectrometer 5973N as the detector. Helium was the carrier gas in the GC system and the column temperature was increased at 7 °C/min between 100–300 °C. Samples were injected using split mode and the total time was 46 min. MS conditions were measured at 70 eV with a mass range of m/z 50–600 amu. Identification of components based on peaks of gas chromatographic analyses was performed through a mass spectrum database search (Wiley 10th edition/NIST 2014 Combined Library).

### 2.6. Morphological Analysis

#### 2.6.1. Scanning Electron Microscopic Study

To observe any ultrastructural changes in the mosquitoes owing to the effects of the six types of extract, scanning electron microscopy was used. The dead mosquitoes were collected from all groups of the adulticidal tests (seven mosquitoes per group) and immersed in a primary fixative with 2.5% glutaraldehyde and a secondary fixative with 1% osmium tetroxide. Next, they were dehydrated in graded ethanol, dried in a critical dryer (HCP-2; HITACHI, Japan) and stubbed and coated with sputter coater (EMITECH K550, Emitech Ltd., Ashford, UK). Fine morphological changes were examined under a scanning electron microscope (model JSM-6610LV, JEOL, Tokyo, Japan).

#### 2.6.2. Histopathological Study

To compare histopathological features in the mosquitoes treated with the six types of essential oils and in untreated mosquitoes, a histopathological analysis was conducted [21]. The mosquitoes were collected and fixed in 10% neutral buffer formalin for 7 days. Standard tissue processing was performed, involving dehydration in graded ethanol, infiltration and embedding in paraffin, and 5-μm thick sectioning and staining by hematoxylin & eosin. Histopathological changes were examined under a light microscope by focusing on compound eyes, thoracic muscles, gastrointestinal tracts, malpighian tubules and ovaries. There were three grades of tissue alteration, including 1 = mild alteration, 2 = moderate alteration and 3 = severe alteration (0 indicated tissue was intact). The severity of the changes was scored using H-score, which was calculated by the multiplication of the severity of their alterations and the percentage of an affected area (0–100%). The scores ranged from 0 to 300.

### 2.7. Statistical Analysis

All quantitative data were described using descriptive statistics in SPSS version 18.0 software (SPSS, Chicago, IL, USA). The average adult mortality was subjected to probit analysis for calculating lethal doses, LD_50_ and LD_90_ at a 95% confidence limit (CL). One-way analysis of variance (ANOVA) was used to determine statistically significant differences between concentrations of six types of essential oils extracted from *A. conyzoides* against adult *Ae. aegypti*. Mean differences were compared using Scheffe’s test after a significant F-test at *p* value < 0.05. Values of *p* < 0.05 were considered significant.

## 3. Results

### 3.1. Plant Extracts

#### Essential Oils and Crude Extracts

Percentage yields obtained from the six types of essential oils (0.09–0.28%) and crude extracts (10.30–17.41%) are shown in Table 1. Crude extract removal using ethanol maceration yielded high percentages. The highest percentage yield for the essential oils and crude extracts were observed in the flower and leaf parts, respectively. The essential oils from the leaves exhibited a lighter yellow colour than those from the flower. The crude extracts exhibited a dark-greenish colour.

### 3.2. Mosquito Tests

#### 3.2.1. Larvicidal Activity

Larvicidal activity screening in the six types of crude extracts using 10 mg/L solutions was performed up to the early fourth instar larvae stage of *Ae. aegypti*. No extract produced considerable effects in this exploration. Therefore, tests for varying concentrations were not conducted.

#### 3.2.2. Adulticidal Activity

**Figure 2:**
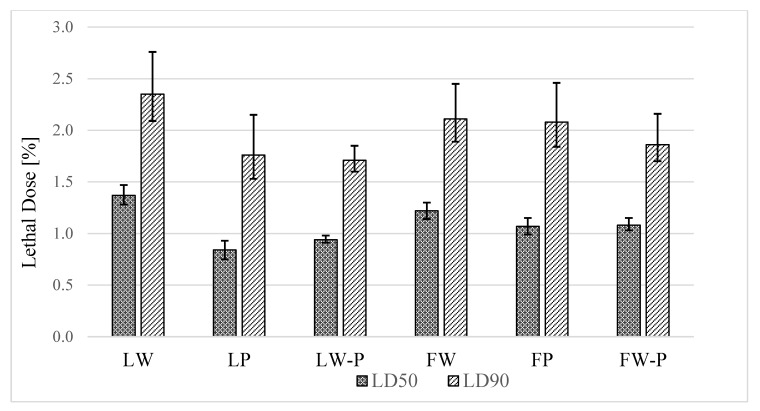
“Lethal dose with 95% CL of six types of *A. conyzoides* essential oils against *Ae. aegypti*. LW: Leaf white, LP: Leaf purple, LW-P: Leaf white-purple, FW: Flower white, FP: Flower purple and FW-P: Flower, white-purple.

Adulticidal activity was investigated for six types of essential oils and seven concentrations of *A. conyzoides* against adult female *Ae. aegypti*. The results indicated that various concentrations of essential oils influenced the mortality of mosquitoes at *p* value < 0.001. Mean mortality rate of mosquitoes increased with increase in concentrations of essential oils with 1.35–2.00% (F = 30.06, *p* < 0.001), 1.05–1.95% (F = 20.88, *p* < 0.001), and 0.55–1.85% (F = 59.52, *p* < 0.001) in essential oil types LW, LP and LW-P, and 1.05%–2.00% (F = 31.61, *p* < 0.001), 0.95–1.95% (F = 62.18, *p* < 0.001) and 0.95–1.85% (F = 59.14, *p* < 0.001) in essential oils types FW, FP and FW-P, respectively (Table 2 and Table 3). No mortality was observed in the control group. After exposure to the test concentrations from the beginning to the end of the experiment, the treated adults exhibited hyperactivity followed by hyperexcitation with rapid progression to knock-down at the bottom of the plastic glass. The highest adult mortalities in female *Ae. aegypti* were observed in LP (LD_50_ = 0.84%) (Table 4 and Figure 2). In addition, the lowest efficiencies were observed in the FW and LW treatments.

### 3.3. GC-MS Analysis

The number of chemical constituents and components in the six types of essential oils extracted from *A. conyzoides* were determined using GC-MS (Table 5). Chemical constituents and components of the six types of essential oils were not similar, even in the same plant parts and in flowers with the same colour. Flowers in each of the three colours had higher numbers of chemical constituents than leaves. The numbers of chemical constituents were also different among the flowers with the three colours. White-purple coloured flowers had a higher number of chemical constituents than the white coloured flowers, followed by the purple coloured flowers. Fourteen chemical components and eight major components were observed, with similarities in the six types of essential oils but differences in their percentages, and six minor components were different with regard to plant parts and colours. Fourteen chemical components were found in 80% of the oils. Chromene was the most common component in the group of six essential oils, followed by sequiterpenes and monoterpenes groups (Table 5). The major components among them were composed of three components including precocene I, β-caryophyllene and precocene II. Precocene I was present in the highest concentration except in one plant type, LP, which had precocene II with the highest concentration. In addition, minor components, including α-caryophyllene, germacrene D, copaene, caryophyllene oxide and 6-vinyl-7-methoxy-2,2-dimethylchromene were observed in the six types of essential oils. Flowers in the three colours had minor components, including α-pinene, camphene, β-pinene and limonene. Notably, endo-bornyl acetate was identified as a minor component in the leaf and flower parts with W-P colour.

### 3.4. Morphological Studies

**Figure 3:**
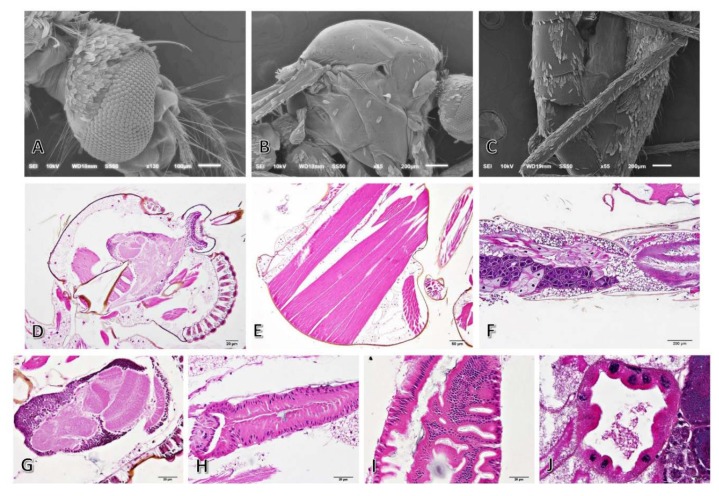
Ultrastructure and histology of mosquitoes with or without six types of essential oil treatment. No major external structural alternations were observed in mosquitoes treated with essential oils compared to untreated mosquitoes. Fine structure of head (**A**), thorax (**B**) and abdomen (**C**) in the mosquitoes subjected to oil extract treatment indicating intact external architecture with general loss of scales Histopathological micrograph of non-treated mosquitoes from head (**D**), thorax (**E**) and abdomen (**F**), which represented the intact compound eyes (**D** and **G**), oesophageal ganglion (**G**), head and thoracic muscle (**D**,**E**) and intestinal tract; anterior, middle and hind gut (**H**, **I** and **J**, respectively).

**Figure 4:**
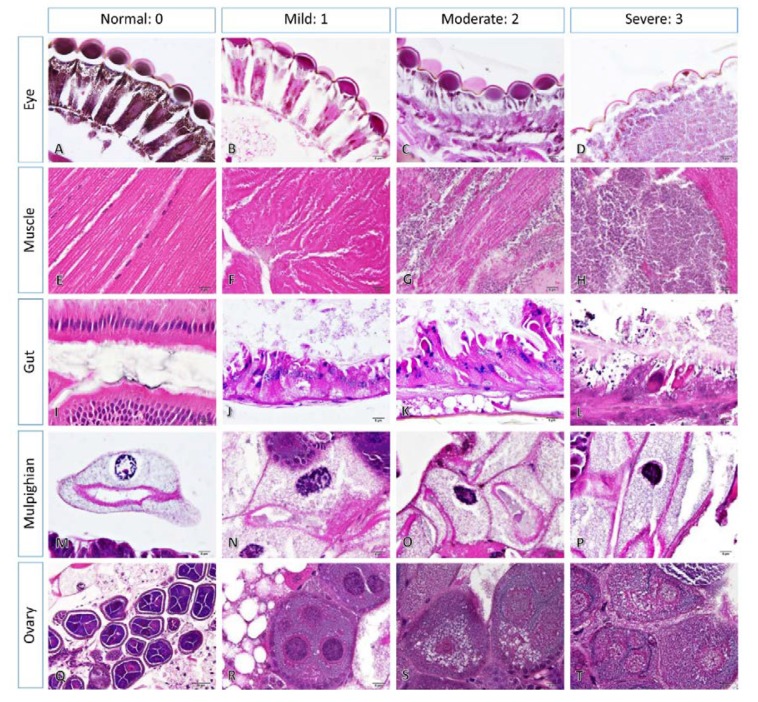
The severity scores of histopathological changes in several tissues of the mosquitoes: Post 24 h of any oil extract exposure, tissue alterations were observed in compound eyes (**A**–**D**), muscles (**E**–**H**), intestinal tracts (**I**–**L**), malpighian tubules (**M**–**P**) and ovaries. Severity ranged from 0–3; 0 = intact, 1–3 = mild, moderate and severe, respectively. Mild to severe alterations of compound eyes were characterised by the following: (+) the deletion and (++) the collapse of retinal and pigmented cell layers with corneal degeneration (**B** and **C**, respectively) and (+++) total destruction of cornea and retina (**D**). Muscular degeneration was indicated by (+) the loss of striated lines without cellular infiltration (**F**), whereas the loss of striated line with moderate (**G**) to severe (**H**) haemocyte infiltration were categorised as (++) and (+++), respectively. Gut lesion was determined by the absence (+) or presence (++) of lipid degeneration with epithelial destruction (**J** and **K**, respectively) and (+++) severe epithelium loss and necrosis. The levels of pyknotic nuclei in the malpighian cell were identified by the increase in density and distribution of heterochromatin; mild (+), moderate (++) and severe (+++), **N** to **P**, respectively. Finally, ovarian degeneration was examined by the distribution of swollen and vacuolated cells; (+) < 25% (**R**), (++) 25%–50% (**S**) and (+++) > 50% (**T**) of observed cells.

Histopathological and scanning electron microscopic studies were conducted to examine the toxicological effects of six types of essential oils on *A. conyzoides* based on the morphological changes in the mosquitoes after 24 h of exposure. The results revealed that the external fine morphologies in the head, thorax and abdomen in the treated mosquitoes were similar to those in mosquitoes without treatment (Figure 3A–C). Loose scales were observed in numerous parts of the mosquitoes. However, histopathological appearances in the mosquitoes with and without treatment varied considerably in terms of severity. Normal architecture of the head, thorax and abdomen is presented in Figure 3D–F, comprising intact compound eyes, head and thoracic muscles, oesophageal ganglions and intestinal tract (Figure 3G–J). Several histopathological alterations attributed to each of the types of oil extract were observed, including compound eyes degeneration, muscular damage with cellular infiltration, gut epithelial degeneration and necrosis, pyknotic nuclei in the malpighian epithelium, and ovarian cell degeneration (Figure 4).

**Figure 5:**
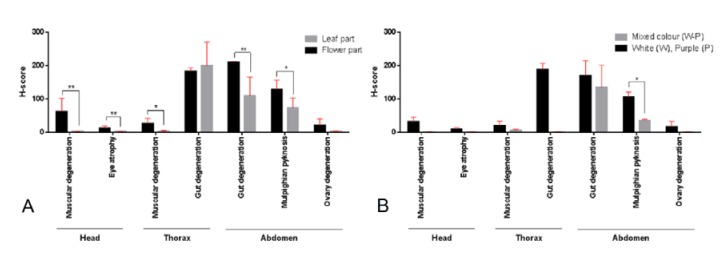
Comparison of histopathological scores as influenced by extracts based on either (**A**) part or (**B**) type of plants; bar graphs exhibit the H-score of any histopathological change between essential oils of leaf and flower parts in white and purple and white-purple or mixed colour of the plants. *: *p* < 0.05, **: *p* < 0.01.

Regarding the severity scores of the histopathological changes, dose-dependent effects were not observed in all kinds of essential oils, except in the malpighian pyknosis associated with the extracts of the LP type (Table 6). Toxicological effects of essential oils from FW and FP plant extracts on mosquito histopathology, as mentioned above, were observed from head, thorax, to abdomen, whereas the rest of the extracts caused lesions that were limited to the abdomen, in the form of gut degeneration and malpighian pyknosis. In particular, essential oils from flower parts contributed highly to the severity of tissue alterations compared with the extracts from leaf parts of all colours (Figure 5A). In addition, the histopathological severity of the essential oils from the white-purple or mixed colour plants tended to be lower than the others (Figure 5B).

## 4. Discussion

The objective of the present study was to provide information about the efficiency of *A. conyzoides* extracts when used against *Ae. aegypti* with regard to lethal effects, chemical constituents and components of essential oils of the plant, and to elucidate the morphological changes in adult female mosquitoes following treatment with the plant extract and essential oils. In the literature, there has been considerable variability in the yields of essential oils from varied plant parts, which was consistent with the findings of the present study (Table 1) [22,23,24]. The highest number of chemical components of five out of six types of essential oils of this plant in Thailand (Table 5) was similar to those reported in previous studies, e.g., in Nigeria, hydrodistillation of fresh leaves and flowers yielded 0.25% *v*/*w* and 0.06%, with the major component as precocene I (57.2% and 82.2%, respectively) [22,25]. Precocene I was reported as the major component in leaves and flowers of *A. conyzoides* in Egypt, Cameroon and Ghana (68.3%, 81% and 83%, respectively) [26,27]. Alternatively, LP extract had precocene II as the predominant compound. Similarly, precocene II (25.89% in South China) was the most abundant in dry leaves and flower parts in South China and dry leaves and stem parts in India, with steam distillation yielding 0.4% (*v*/*w*) and 0.1% oil [24,28]. Chemical constituents and major components of essential oils can vary, not only owing to numerous environmental factors such as climates, seasons, soil compositions, plant organs, ages, harvesting times, nutritional status and method of extraction, but also by region [29,30].

One of the most critical aspects of plant extract activity is efficiency against insects. Plant extracts have been reported to have potential uses in controlling vectors or pests in storage systems [24,31,32,33]. The present study investigated the efficiency of *A. conyzoides* in the form of crude extracts and essential oils. Six types of crude extract were not effective against larval stages of *Ae. aegypti* following exposure for 48 h. Similarly, dried aerial parts of *A. conyzoides* extracted using 95% and 75% ethanol maceration exhibited no toxicity to larvae of *Ae. fluviatilis* and *Ae. aegypti* at 100 and 1600 mg/L doses, respectively [34,35]. Moreover, previous studies have reported that absolute and 95% ethanol extracts could have antidiarrheal and antidiabetic properties in albino rat models and against field ticks in India [24,36]. The varying degrees of toxicity of crude extracts against either insects or diseases due to numerous constituents of natural products have attracted special interest. A wide variety of biological properties are exhibited by most crude extracts. Some studies have used essential oils and methanol extracts for controlling vectors at larval stages, and observed that essential oil extracts were more effective than methanol extracts [37]. Essential oils are volatile compounds, which are not suitable for use in controlling vectors in breeding sites, however, essential oil extracts from *Pinus kesiya* and *Zanthoxylum monophyllum* act as potential larvicides against mosquito vectors [33,38]. The toxic effects of *Piper sarmentosum*, *P. ribesoides* and *P. longum* by topical application against *Ae. aegypti* with LD_50_ were 0.14, 0.15 and 0.26 µg/female, respectively, and LD_50_ of *Apium graveolens* were 6.6 mg/cm^2^ impregnated paper [39,40]. The efficiency of essential oils extracted from *Lantana camara* was 0.06 mg/cm^2^ by oil-impregnated paper [41]. In addition to the different techniques, there are differences in the units used to report the results of adulticidal activity, which may pose challenges for the comparison of efficiency among plants. Regarding adulticidal activity using essential oils, six types of essential oils of *A. conyzoides* were effective against adult female *Ae. aegypti* (Table 4 and Figure 2). However, it is challenging to evaluate the efficiency against mosquitoes, as previous studies have not reported the adulticidal activity of the plant against mosquitoes through topical application. The percentage yield of the six types of essential oils were quite low, particularly from leaves, which may be one reason why oils from *A. conyzoides* had not been studied against adult mosquitoes via topical application. Nevertheless, in the present study, the efficiency of the plant extracts and essential oils could be evaluated from the LD_50_ and LD_90_. Hence, this study presents the first investigation on *Ae. aegypti* mosquitoes. Extracts from LW and FW plants exhibited the lowest efficiency, whereas those from LP exhibited the highest efficiency against mosquitoes, potentially associated with the highest levels of precocene II reported in LP. Low or high activity against vectors is not the most critical factor when using extracts from natural products to control vectors. It is essential to use plant products that exhibit low toxicity to mammals and are biodegradable [8,42]. Essential oils of *A. conyzoides* are potential biological insecticide candidates, and the chemical components of six types of essential oils were explored to provide information on the insecticidal effects.

According to the results from the chemical component analyses, the six types of essential oils belong to various chemical groups. The predominant chemical group represented in these six essential oils was chromene (e.g., precocene I and precocene II). This group has been shown to have antijuvenile hormonal effects, which is probably responsible for the insecticidal effects, such as antigonadotropic effects, ovicidal effects, precocious metamorphosis and diapause induction in insects [16]. Sequiterpenes and monoterpenes were minor groups in the essential oils. The structures and functions of the sequiterpene group are similar to those of the monoterpene group. Monoterpenes rapidly penetrate insects and interfere with their physiological functions. Investigating the mode of action of monoterpenes is complex [43]. However, these three chemical groups of essential oils are known to have insecticidal properties [44,45]. Contact of the essential oils with the thorax of mosquitoes triggered hyperactivity, followed by hyperexcitation and rapid knock-down at the bottom of the plastic glasses, which suggests a neurotoxic action [46]. The symptoms could be due to monoterpenes, as they have been shown to produce neurotoxicity in insects [47]. Regarding the toxicity of the six types of essential oils to the external and internal organs of an adult mosquito, no previous studies have been reported in the literature. External morphology did not change with or without the six types of essential oil treatments (Figure 3A–C). Therefore, the chemical components of the essential oils did not affect the external morphology of the mosquitoes. However, histopathological features in the internal architecture of mosquitoes were observed in many parts (Figure 3). According to the severity scores of the histopathological changes, dose-dependent effects were observed only in LP plant type, which caused malpighian pyknosis (Table 6). Malpighian tubules are the primary organ in insects used for excretion and osmoregulation [48]. The condensation of chromatin and nucleus in malpighian pyknotic cells may result from apoptosis; programmed cell death. Agricultural contamination by imidacloprid adversely affects non-target organs such as mulpighian tubules, for example the malpighian tubules of *Apis mellifera*, causing increased cell apoptosis as highlighted by De Almeida Rossi et al. [49].

The biological activity of essential oils is largely attributed to their major components [50]. Although in some cases, major components may not be responsible for the overall activity, the presence of a combination of major and minor components could have resulted in additive, synergistic, or antagonistic interactions [50]. The present study demonstrated high levels of tissue alteration in the mosquitoes treated with essential oils from flower parts, as they contain α-pinene, camphene, β-pinene and limonene, which belong to the monoterpenes group (Table 5). All the above-mentioned terpenoids were not the major components of the extracts. The severity of tissue alterations was observed to be low when treated with extracts from mixed colour plants and leaf parts, probably owing to the presence of endo-bornyl acetate and β-bourbonene, respectively (Figure 5 and Table 5). Nevertheless, further studies are required to explore their effects.

## 5. Conclusions

This is the first study on the efficiency of extracts from different plant parts and different-coloured parts of *A. conyzoides* against larva and adult stages of *Ae. aegypti* mosquitoes. Crude extracts did not exhibit any larvicidal activity. However, six types of essential oils, especially from the LP extract, exhibited effective adulticidal activity due to the constituents, including precocene II, which was the major component. The monoterpene group could be responsible for the histopathological alterations in the mosquitoes. This could explain the difference in tissue alteration observed between the leaf and flower parts of *A. conyzoides*. The chemical constituents and components of six types of essential oils were explored in this study to provide insecticidal data on the plant and to validate the components in subsequent steps. Our results suggest that the plant extracts of *A*. *conyzoides* could be used as a biological insecticide for vector control.

## Figures and Tables

**Table 1:** Six types of plants and percent (%) yield of essential oils and crude extracts from *A. conyzoides.*

No.	Plant Types	Plant Parts	Colour	Represents	Essential Oils (%)	Crude Extracts (%)
1	LW	Leaf	White	Leaf white	0.17	17.41
2	LP	Leaf	Purple	Leaf purple	0.09	11.44
3	LW-P	Leaf	White-Purple	Leaf white-purple	0.19	12.15
4	FW	Flower	White	Flower white	0.17	10.30
5	FP	Flower	Purple	Flower purple	0.16	12.24
6	FW-P	Flower	White-Purple	Flower white-purple	0.28	10.95

**Table 2:** Mean mortality rate of adulticidal activity of essential oils extracted from leaf parts of *A. conyzoides* with three colours against adult *Ae. aegypti* females.

Plant Types	Concentrations (%)	n	Mean	SD	F	*p* Value
LW	0.70	180	2.33 ^A^	2.08	30.06	<0.001 *
	0.80	180	6.33	2.52		
	0.90	180	10.00	6.08		
	1.05	180	20.00	4.36		
	1.35	180	26.00 ^A^	8.72		
	1.75	180	40.67 ^A^	7.77		
	2.00	180	51.33 ^A^	5.51		
LP	0.40	180	6.67 ^B^	3.79	20.88	<0.001 *
	0.65	180	22.00	10.15		
	0.95	180	27.67	10.02		
	1.05	180	40.00 ^B^	7.00		
	1.35	180	48.00 ^B^	7.21		
	1.75	180	54.67 ^B^	4.04		
	1.95	180	56.67 ^B^	3.21		
LW-P	0.55	180	7.00 ^C^	3.46	59.52	<0.001 *
	0.65	180	13.67 ^C^	3.79		
	0.85	180	24.00 ^C^	3.46		
	0.95	180	31.33 ^C^	2.52		
	1.05	180	34.00 ^C^	3.00		
	1.45	180	48.33 ^C^	7.23		
	1.85	180	56.67 ^C^	2.31		

* *p* < 0.05 is significant.A, B, and C = Mean mortality rate of mosquitoes increased with increase in concentrations of essential oils; LW, LP, and LW-P, respectively (*p* < 0.001).

**Table 3:** Mean mortality rate of adulticidal activity of essential oils extracted from flower parts of *A. conyzoides* with three colours against adult *Ae. aegypti* females.

Plant Types	Concentrations (%)	n	Mean	SD	F	*p* Value
FW	0.70	180	5.67 ^D^	1.53	31.61	<0.001 *
	0.80	180	8.00	3.61		
	0.90	180	13.67	6.43		
	1.05	180	26.33 ^D^	6.81		
	1.35	180	37.67 ^D^	7.37		
	1.75	180	44.67 ^D^	6.03		
	2.00	180	53.33 ^D^	6.35		
FP	0.45	180	5.67 ^E^	5.51	62.18	<0.001 *
	0.65	180	9.00	2.65		
	0.95	180	20.33 ^E^	5.51		
	1.05	180	29.33 ^E^	4.93		
	1.35	180	37.67 ^E^	5.51		
	1.75	180	51.00 ^E^	2.00		
	1.95	180	55.33 ^E^	1.15		
FW-P	0.55	180	5.00 ^F^	3.46	59.14	<0.001 *
	0.65	180	8.00	3.00		
	0.85	180	17.67	3.06		
	0.95	180	21.00 ^F^	3.00		
	1.05	180	24.67 ^F^	5.51		
	1.45	180	43.00 ^F^	6.93		
	1.85	180	57.00 ^F^	2.65		

* *p* < 0.05 is significant. D, E, and F = Mean mortality rate of mosquitoes increased with increase in concentrations of essential oils; FW, FP, and FW-P, respectively (*p* < 0.001).

**Table 4:** Lethal doses, LD_50_ and LD_90_ of six types of essential oils of *A. conyzoides* against adult, female *Ae. aegypti.*

Plant Types	n	Slope ± SE	LD_50_ [%] (95% CL)	LD_90_ [%] (95% CL)	χ^2^
LW	1260	5.44 ± 0.28	1.37 (1.28–1.47)	2.35 (2.09–2.76)	55.09
LP	1260	3.40 ± 0.21	0.84 (0.75–0.93)	1.76 (1.53–2.15)	77.61
LW-P	1260	4.99 ± 0.27	0.94 (0.91–0.98)	1.71 (1.60–1.85)	27.32
FW	1260	5.36 ± 0.27	1.22 (1.14–1.30)	2.11 (1.89–2.45)	56.06
FP	1260	4.42 ± 0.27	1.07 (0.99–1.15)	2.08 (1.84–2.46)	55.22
FW-P	1260	5.33 ± 0.28	1.08 (1.03–1.15)	1.86 (1.70–2.16)	41.04

95% CL = 95% confidence limits; n = total number of mosquito samples; χ^2^ = chi-square.

**Table 5:** The chemical constituents and components of six types of essential oils of *A. conyzoides*, expressed as percent of total area.

No.	Components	Essential Oils Groups	Plant Types (Number of Constituents)					
			LW (30)	LP (24)	LW-P (32)	FW (36)	FP (35)	FW-P (42)
1	Precocene I	Chromene	61.32	13.99	48.04	36.15	42.86	25.76
2	β-caryophyllene	sesquiterpenes	20.09	24.17	20.60	22.86	21.49	24.67
3	Precocene II	Chromene	0.87	47.49	12.81	14.45	14.45	15.96
4	α-caryophyllene	sesquiterpenes	2.36	1.39	2.51	5.09	4.09	6.62
5	Germacrene D	sesquiterpenes	2.98	1.48	2.87	3.79	3.46	5.64
6	Copaene	sesquiterpenes	0.20	0.11	0.21	0.37	0.08	0.52
7	Caryophyllene oxide	sesquiterpenes	0.99	0.87	0.58	0.58	0.43	0.45
8	6-vinyl-7-methoxy-2,2-dimethylchromene	Chromene	0.28	0.39	0.17	0.17	0.17	0.28
9	α-pinene	monoterpenes				0.12	0.06	0.10
10	Camphene	monoterpenes				0.93	0.93	0.58
11	β-pinene	monoterpenes				0.11	0.05	0.06
12	Limonene	monoterpenes				0.34	0.14	0.18
13	β-bourbonene	sesquiterpenes	0.12	0.12	0.14			
14	endo-bornyl acetate	monoterpenes			0.17			0.55

**Table 6:** Histopathological scores in the mosquitoes treated with the six types of essential oils of *A. conyzoides* and in those that went untreated.

Histopathology Mean (SEM)	Plant Type LW			Plant Type LP			Plant Type LW-P			Plant Type FW			Plant Type FP			Plant Type FW-P		
	Low [0.70%]	Med [1.05%]	High [2.00%]	Low [0.4%]	Med [1.05%]	High [1.95%]	Low [0.55%]	Med [0.95%]	High [1.85%]	Low [0.7%]	Med [1.05%]	High [2.00%]	Low [0.45%]	Med [1.05%]	High [1.95%]	Low [0.55%]	Med [0.95%]	High [1.85%]
Head																		
Muscular degeneration	0	0	0	0	0	0	0	0	0	100.0	30.0	0	160.0	0	0	0	0	0
	(0)	(0)	(0)	(0)	(0)	(0)	(0)	(0)	(0)	(50.0)	(30.0)	(0)	(47.6)	(0)	(0)	(0)	(0)	(0)
Eye atrophy	0	0	0	0	0	0	0	0	0	33.3	20.0	0	30.0	0	0	0	0	0
	(0)	(0)	(0)	(0)	(0)	(0)	(0)	(0)	(0)	(16.6)	(20.0)	(0)	(15.2)	(0)	(0)	(0)	(0)	(0)
Thorax																		
Muscular degeneration	0	0	0	0	0	0	4.2	10.0	0	100.0	30.0	0	32.0	3.7	0	9.0	0	0
	(0)	(0)	(0)	(0)	(0)	(0)	(4.2)	(6.3)	(0)	(50.0)	(30.0)	(0)	(16.2)	(3.7)	(0)	(4.5)	(0)	(0)
Gut degeneration	0	300.0	166.6	0	0	0	0	0	0	225.0	225.0	118.7	0	300.0	200.0	0	0	0
	(0)	(0)	(88.2)	(0)	(0)	(0)	(0)	(0)	(0)	(75.0)	(47.8)	(68.7)	(0)	(0)	(100.0)	(0)	(0)	(0)
Abdomen																		
Gut degeneration	116.6	0	130.0	237.5	90.0	300.0	0	0	0	271.4	225.0	114.0	287.5	280.0	200.0	300.0	160.0	100.0
	(47.7)	(0)	(77.2)	(47.3)	(24.4)	(0)	(0)	(0)	(0)	(18.4)	(47.8)	(35.2)	(12.5)	(12.2)	(100.0)	(0)	(40.0)	(100.0)
Ovarian degeneration	0	0	0	0	0	0	0	0	0	150.0	0	0	0	0	0	0	0	0
	(0)	(0)	(0)	(0)	(0)	(0)	(0)	(0)	(0)	(67.1)	(0)	(0)	(0)	(0)	(0)	(0)	(0)	(0)
Malpighian pyknosis	116.6	0	130.0	0 *	166.0 *	240.0 *	55.7	0	0	204.0	75.0	204.0	52.5	137.1	111.6	60.8	0	95.0
	(47.7)	()	(77.2)	(0)	(46.6)	(30.0)	(32.3)	(0)	(0)	(61.7)	(56.7)	(61.7)	(49.2)	(51.9)	(54.0)	(12.6)	(0)	(65.0)

* Significant differences among low, medium and high doses.
